# Tight species cohesion among sympatric insular wild gingers (*Asarum* spp. Aristolochiaceae) on continental islands: Highly differentiated floral characteristics versus undifferentiated genotypes

**DOI:** 10.1371/journal.pone.0173489

**Published:** 2017-03-16

**Authors:** Junshi Matsuda, Yoshiyuki Maeda, Junichi Nagasawa, Hiroaki Setoguchi

**Affiliations:** 1 Graduate School of Human and Environmental Studies, Kyoto University, Yoshida Nihonmatsu-cho, Sakyo-ku, Kyoto, Japan; 2 Faculty of Science, Kagoshima University, Kagoshima, Japan; 3 Kyoto Botanical Garden, Shimokamo Hangi-cho, Sakyo-ku, Kyoto, Japan; National Cheng Kung University, TAIWAN

## Abstract

The Amami Island group of the Ryukyu Archipelago, Japan, harbors extensive species diversity of *Asarum* in a small landmass. The fine-scale population genetic structure and diversity of nine insular endemic *Asarum* species were examined using nuclear DNA microsatellite loci and ITS sequences. High population genetic diversity (*H*_S_ = 0.45–0.79) was estimated based on the microsatellites, implying outcrossing of *Asarum* species within populations accompanied by inbreeding. Bayesian clustering analyses revealed that species were divided into three robust genetic clusters and that the species within each cluster had a homogeneous genetic structure, indicating incomplete lineage sorting. This conclusion was supported by an ITS phylogeny. The degree of genetic differentiation among species was very low both within and between clusters (*F*_ST_ = 0.096–0.193, and 0.096–0.266, respectively). Although species can be crossed artificially to produce fertile hybrids, our results indicate that there is very little evidence of hybridization or introgression occurring among species in the wild, even within stands composed of multiple sympatric species. The highly differentiated floral morphology of the studied species is likely to impose reproductive isolation between them and maintain their integrity in the wild. A lack of genetic differentiation between sympatric species suggests that speciation within this group occurred rapidly and recently.

## Introduction

The species richness of islands makes them suitable experimental systems for elucidating speciation mechanisms. Traditional island biogeography studies [1, 2] have suggested that this richness is due mostly to the speciation of migrants within and among islands, and recent island phylogeography studies have corroborated this conclusion [3–6]. Insular speciation, particularly its initial stages, principally occurs among geographically distinct populations (i.e., allopatric or parapatric speciation) [7–11]. However, recent studies have suggested that parapatric or sympatric speciation can occur within a relatively small area on an island [12–14]. The small landmass of islands may prompt insular endemics to make secondary contact, causing gene exchange between species via hybridization and introgression [6, 15, 16]. In this case, the integrity of each species might become reduced or disappear at the morphological and/or molecular levels. Maintenance of species cohesion is especially important for understanding the rich biodiversity of island ecosystems, which frequently harbor species assemblages that are products of recent adaptive radiation and sympatric or parapatric occurrence.

Reproductive isolation in sympatric zones has attracted the interest of many evolutionary biologists. Previous studies have suggested that pre- and postzygotic isolation are important for reproductive isolation between sympatric sister species [17–19], while recent studies have shown that prezygotic isolation can be more important than postzygotic isolation [20–22]. For example, floral isolation such as through pollinator behavior (ethology) or mechanical isolation of flowers can be an important prezygotic barrier in which the divergence of floral morphology and species-specific pollinators have contributed to reproductive isolation among species [23–28]. Another explanation for reproductive isolation is ecological speciation in which habitat differences generate reproductive barriers between populations of a species, potentially leading to speciation [19, 29–32]. Consequently, the species richness of island floras could be maintained by either one or multiple mechanisms of reproductive isolation.

**Fig 1:**
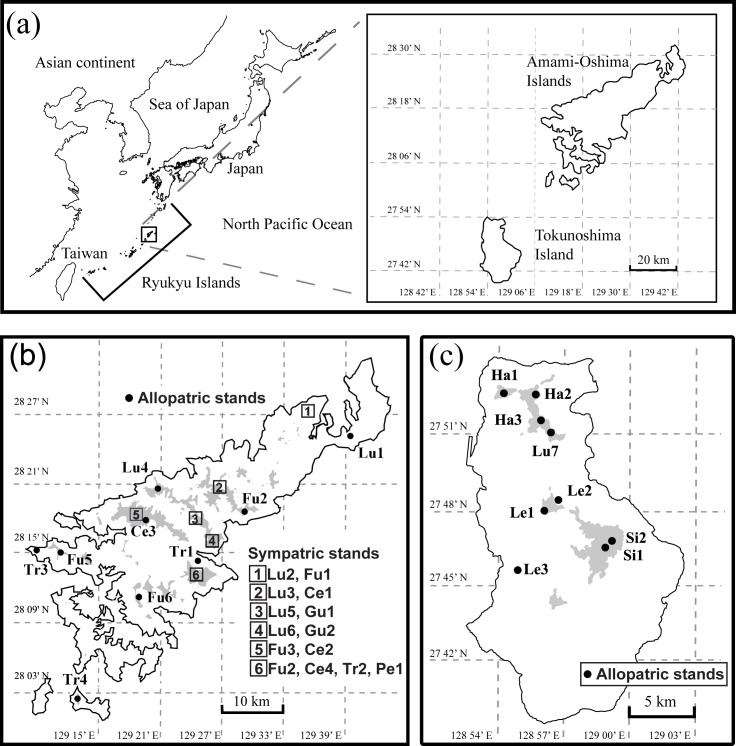
Map showing the location of the sampling sites. (a) area map with the Ryukyu Archipelago positioned centrally; (b) Amami-Oshima Island; (c) Tokunoshima Island. Dark gray regions indicate altitudes exceeding 350 m; closed circles denote allopatric stands; squares represent sympatric stands.

The genus *Asarum* L. (Aristolochiaceae) section *Heterotropa* Morr. et Dence (Wild ginger) consists of perennial evergreen herbs that grow in shaded understory and exhibit high floral divergence among species. The section includes approximately 72 species in East Asia [33, 34] and is very diverse in Japan, where 46 species are recognized. Nineteen of these species are confined to the Ryukyu Archipelago, with most recognized as insular endemics to a particular island [34, 35]. Notably, nine of the 19 species are confined to the small landmass of the Amami Group (Amami-Oshima and Tokunoshima islands) located in the center of the Ryukyu Archipelago (Fig 1).

Both Amami-Oshima and Tokunoshima islands are mountainous (highest altitudes 694 and 645 m, respectively), with areas of 712 and 248 km^2^, respectively (Fig 1B and 1C). These islands have been connected several times in the past by land-bridges ascribed to glacial regression during the Pleistocene [36–40]. Therefore, *Asarum* species might have had opportunities to migrate across the islands during glacial periods, but would have become isolated on each island during the inter- and postglacial periods. *Asarum* seed dispersal might be aided by small insects (via the aril appendage), and in the case of *Asarum takaoi* F. Maek. in mainland Japan, ants were shown to disperse seeds over distances of <50 cm/year [41]. Among the nine *Asarum* species distributed in the Amami Group, *Asarum lutchuense* T.Ito ex Koidz. inhabits both Amami-Oshima and Tokunoshima, indicating past migration and/or continuous land configuration via a landbridge. In contrast, the remaining eight species are endemic to one island: five species (*Asarum fudsinoi* T.Itô, *Asarum celsum* F. Maek. ex Hats. & Yamahata, *Asarum gusk* Hats. & Yamahata, *Asarum pellucidum* Hats. & Yamahata, *Asarum trinacriforme* Hats. & Yamahata) are endemic to Amami-Oshima, and three (*Asarum hatsushimae* F. Maek. ex Hats. & Yamahata, *Asarum leucosepalum* Hats. & Yamahata, *Asarum simile* Hats. & Yamahata) are endemic to Tokunoshima, possibly due to speciation within each island. The distribution pattern on each island differs: the five species (including *A*. *lutchuense*) on Amami-Oshima frequently grow in sympatric stands (see S1 Fig), whereas species on Tokunoshima Island are allopatric (see Fig 1, Table 1).

**Table 1:** Sample information for 32 populations of Asarum on Amami Group.

Species	Island	Locality	Population	Stand		Number of	Latitude	Longitude	Altitude
			name	Situation	Number of sympatric stand*	individuals sampled	(N)	(W)	(m)
*Asarum lutchuense*	Amami-Oshima	Itton	Lu1	allopatric		32	28°25´333´´	129°39´594´´	10
	Amami-Oshima	Tatsugou	Lu2	sympatric	1	36	28°27´181´´	129°35´510´´	265
	Amami-Oshima	Kinsakubaru	Lu3	sympatric	2	30	28°20´255´´	129°26´725´´	450
	Amami-Oshima	Ogawatake	Lu4	allopatric		10	28°20´073´´	129°20´246´´	380
	Amami-Oshima	Sutarumata	Lu5	sympatric	3	19	28°17´304´´	129°25´087´´	430
	Amami-Oshima	Takinohana	Lu6	sympatric	4	31	28°15´739´´	129°26´147´´	420
	Tokunoshima	Sasontuji	Lu7	allopatric		39	28°50´797´´	129°56´742´´	310
*A*. *fudsinoi*	Amami-Oshima	Tatsugou	Fu1	sympatric	1	11	28°27´181´´	129°35´510´´	265
	Amami-Oshima	Asado	Fu2	sympatric	6	31	28°18´450´´	129°28´841´´	360
	Amami-Oshima	Yuwan	Fu3	sympatric	5	30	28°17´766´´	129°19´263´´	700
	Amami-Oshima	Torigamine	Fu4	allopatric		32	28°13´047´´	129°24´224´´	430
	Amami-Oshima	Tokurayama	Fu5	allopatric		32	28°15´044´´	129°10´046´´	320
	Amami-Oshima	Yuidake	Fu6	allopatric		32	28°11´000´´	129°18´805´´	490
*A*. *celsum*	Amami-Oshima	Kinsakubaru	Ce1	sympatric	2	18	28°20´255´´	129°26´725´´	450
	Amami-Oshima	Yuwan 1	Ce2	sympatric	5	29	28°17´878´´	129°19´300´´	700
	Amami-Oshima	Yuwan 2	Ce3	allopatric		21	28°18´294´´	129°18´801´´	700
	Amami-Oshima	Torigamine	Ce4	sympatric	6	16	28°13´088´´	129°24´162´´	450
*A*. *gusk*	Amami-Oshima	Sutarumata	Gu1	sympatric	3	24	28°17´304´´	129°25´087´´	430
	Amami-Oshima	Takinohana	Gu2	sympatric	4	20	28°15´739´´	129°26´147´´	420
*A*. *pellucidum*	Amami-Oshima	Torigamine	Pe1	sympatric	6	5	28°13´088´´	129°24´162´´	450
*A*. *trinacriforme*	Amami-Oshima	Yanma	Tr1	allopatric		29	28°14´008´´	129°24´680´´	270
	Amami-Oshima	Torigamine	Tr2	sympatric	6	30	28°13´088´´	129°24´162´´	450
	Amami-Oshima	Nishikomi	Tr3	allopatric		33	28°15´025´´	129°09´123´´	260
	Amami-Oshima	Ukejima	Tr4	allopatric		19	28°02´134´´	129°13´037´´	280
*A*. *hatsushimae*	Tokunoshima	Amagi_west	Ha1	allopatric		29	27°52´444´´	128°54´819´´	350
	Tokunoshima	Amagi_east	Ha2	allopatric		23	27°52´234´´	128°56´066´´	400
	Tokunoshima	Sasontuji	Ha3	allopatric		32	27°51´362´´	128°56´032´´	360
*A*. *leucosepalum*	Tokunoshima	Minata_west	Le1	allopatric		17	27°47´800´´	128°56´273´´	340
	Tokunoshima	Minata_east	Le2	allopatric		32	27°48´176´´	128°56´562´´	400
	Tokunoshima	Nishiagina	Le3	allopatric		32	27°45´709´´	128°55´119´´	150
*A*. *simile*	Tokunoshima	inokawa_west	Si1	allopatric		32	27°46´660´´	128°58´976´´	630
	Tokunoshima	Inokawa_east	Si2	allopatric		32	27°46´788´´	128°59´179´´	630

* Number of sympatric stand corresponds to those in Fig 1(B)

**Fig 2:**
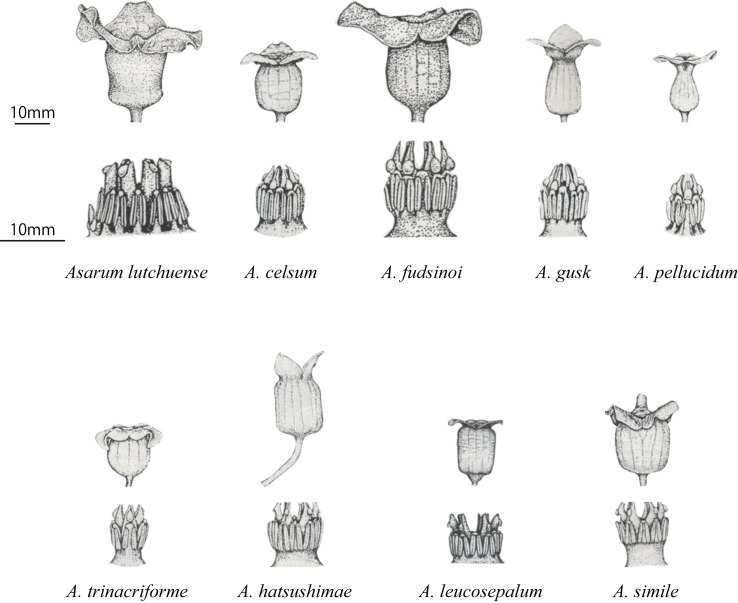
Line drawings of the nine *Asarum* species showing their flowers and reproductive organs.

The floral morphology of the nine endemics is divergent in terms of size, color, calyx tube shape, calyx tube mouth size (throat), numbers of stamens and carpels, and pedicel length [35] (see S1 Table, Fig 2). For example, *A*. *hatsushimae* and *A*. *leucosepalum* develop long pedicels and flowers are arranged above ground, whereas the flowers of other species bloom on the ground. *Asarum gusk* and *A*. *pellucidum* produce small and narrow urceolate flowers with a constricted calyx tube mouth less than 2 mm in diameter. However, *A*. *gusk* has 12 stamens and six carpels, while *A*. *pellucidum* has six stamens and three carpels. Few individuals in stands of sympatric species exhibit intermediate morphology, suggesting that interspecific hybridization is rare. This is despite the fact that all species in the group have identical chromosome numbers (2n = 24) and may be crossed artificially with other species in the Amami Group to produce fertile hybrids [42].

In this study we examined the population genetic structure of the nine *Asarum* species that occur on the Amami-Oshima and Tokunoshima Islands in the Ryukyu Archipelago using nuclear DNA microsatellite (nSSR) loci. We also investigated their phylogenetic relationships based on internal transcribed spacer (ITS) sequence variation. The aims of the study were (i) to determine whether each of the nine insular endemic species are genetically differentiated from each other despite their small habitat area and/or sympatric occurrence; (ii) to determine the degree of inbreeding and outcrossing of species, levels of gene flow, and occurrence of recent genetic bottlenecks; and (iii) to determine whether geographic isolation could be responsible for the genetic differences within a species. We also discuss the evolutionary history of the nine *Asarum* species and propose the factors involved in the formation of reproductive isolating barriers that maintain distinct species boundaries.

## Materials and methods

### Sampling for ITS phylogenetic analysis

To examine the phylogeny of the nine endemics of the Amami Group, we analyzed their ITS sequences together with those of other species from the Ryukyu Islands and mainland Japan, Taiwan, and mainland China. Leaf samples were collected from plants growing in natural habitats and/or the cultivation stocks of botanical gardens. ITS sequences were generally obtained by direct sequencing of PCR products, although some ITS sequences were also identified by TA-cloning using the pT7 Blue T-Vector (Merck, Darmstadt, Germany) and competent cells (*Escherichia coli* JM109: Takara Bio, Otsu, Japan) if a heterozygous sequence was inferred. *Asarum caudigerum* Hance was used as outgroup, based on a previously constructed ITS phylogeny for *Asarum* [43]. Sample information of taxa and accession numbers of individuals sequenced are presented in S3 Table.

### Locations and species sampled

*Asarum* stands occur on inselbergs within the mountainous islands of Amami-Oshima and Tokunoshima. We studied two types of stand: one comprising a single species (allopatric stands) and the other comprising multiple *Asarum* species (sympatric stands). The locality and species composition of each stand are summarized in Fig 1 and Table 1. The sympatric stands were confined to Amami-Oshima, whereas all four allopatric stands were distributed on Tokunoshima (Fig 1, Table 1). Our initial aim was to collect leaf samples from more than 30 individuals from each population per species; however, we sampled leaves from less than 30 individuals in 15 of the 32 populations. Individuals were sampled along transects at *c*. 5-m intervals. Only five individuals from one population of *A*. *pellucidum* (an endemic local to Mt. Torigamine on Amami-Oshima) were sampled due to this species being almost extinct in the wild. In total, 838 individuals from 32 populations were used in this study (Table 1). Permissions for collecting samples were obtained from municipal government of Kagoshima.

### DNA extraction, PCR amplification, and microsatellite genotyping

Dried leaf material was pulverized with a TissueLyser (Qiagen, Hilden, Germany), according to the manufacturer’s instructions. After polysaccharides were removed from the powder using HEPES buffer (pH 8.0) [44], DNA was extracted using the cetyltrimethylammonium bromide (CTAB) method [45]. Extracted DNA was dissolved in 100 μL of TE buffer and used for subsequent polymerase chain reaction (PCR) amplification.

For the ITS region, extracted DNA from collected samples (Table 1) was used for direct sequencing of the PCR products. PCR was conducted in a total reaction volume of 25 μL containing 18.5 μL of autoclaved ion-exchanged water, 0.2 mM dNTP mixture, 2.0 mM 10× Ex Taq Buffer (Ex Taq; Takara Bio), 0.625 U Takara Ex Taq (Takara Bio), 0.2 mM of each primer, and 1.25 μL of DNA. PCR was performed for 35 cycles under the following conditions using primers ITS4 and ITS5 [46]: 1 min at 94°C, 1 min at 48°C, and 1 min at 72°C. PCR products were sequenced in both directions using the standard method of the BigDye Terminator Cycle Sequencing Kit ver. 3.1 (Applied Biosystems, Foster City, CA) using the same primers as above on an ABI 3130 Genetic Analyzer (Applied Biosystems). All sequences were analyzed and aligned with AutoAssembler (Applied Biosystems). Insertions and deletions were treated as missing data and excluded from the genotyping and phylogenetic analysis.

The genotypes of 838 individuals were determined for 12 nSSR markers developed for this study (Af-2, Af-20, Af-39, Af-69, Af-116, Af-119, Af-124, Af-125, Af-130, Af-132, Af-144, and Af-147) [47]. Because three (Af-119, Af-124, and Af-125) of the 12 primer pairs for these loci did not cross-amplify in *A*. *lutchuense*, we primarily focused on the other nine loci when analyzing the other endemic species. These nine loci that could be amplified in all of the studied species are henceforth referred to as the core loci.

PCR amplification was performed in a final volume of 6 μL (containing 40–60 ng of genomic DNA) following the standard protocol of the Qiagen Multiplex PCR Kit. Compound SSR primers [(AC)_6_(AG)_5_ or (TC)_6_(AG)_5_] were labeled with the fluorochromes 6-FAM or HEX (Applied Biosystems). The amplification profile was as follows: initial denaturation at 95°C for 15 min followed by 30 cycles of 94°C for 30 s, 58°C (the annealing temperature of the primer pair) for 90 s, and 72°C for 60 s, with a final extension at 60°C for 30 min. The amplified products were loaded onto an ABI 3100 autosequencer (Applied Biosystems) using the GeneScan Rox-350 Size Standard (Applied Biosystems). About 5% of all samples were amplified and genotyped at least twice, and the genotyping error rate was less than 5% for each locus.

### ITS phylogenetic analysis

Sequence data were edited and assembled using ClustalX ver. 1.83 [48]. Sequences were easily aligned with few insertions/deletions (indels), which were removed from the data. Phylogenetic analyses were conducted using MrBayes ver. 3.2.0 [49] for Bayesian inference (BI). The BI analysis was performed using four incrementally heated Markov chains (one cold, three heated) simultaneously started from random trees, and run for 10,000 cycles sampling a tree every 100 generations. The stationary phase was reached when the average standard deviation of split frequencies approached 0.01. Trees that preceded the stabilization of the likelihood value (the burn-in) were discarded, and the remaining trees were used to calculate a majority-rule consensus phylogram. This was viewed and edited using TreeView [50]. The Bayesian posterior probabilities (BPP) for the consensus tree’s internal nodes were shown above the corresponding node.

### Analyses of genetic diversity

The number of alleles, allelic richness [51], observed heterozygosity (*H*_O_), gene diversity, expected within-population heterozygosity (*H*_S_)[52], and inbreeding index (*F*_IS_ = 1 –*H*_O_/*H*_E_) were calculated for each nSSR locus and each population using FSTAT ver. 2.9.3.2 [53]. Departures from Hardy–Weinberg equilibrium (HWE) at each locus and linkage disequilibrium between loci were also tested using FSTAT (alleles were randomized 1000 times over all samples). The number of private alleles for each locus was calculated using GENALEX 6.1 [54]. Significant differences at each locus were tested by the log-likelihood (G)-based exact test [55] using a Markov chain Monte Carlo (MCMC) method implemented in GENEPOP ver. 4.0.10 [56, 57]. Departures from HWE at each locus and within each population were tested with an exact test using the MCMC method implemented within GENEPOP. These analyses were conducted using the nine core loci that were available for amplification in all species.

### Genetic differentiation and population genetic structure

For the nine core nSSRs, the coefficient of genetic differentiation among populations (*F*_ST_)[58] was estimated using fstat. The significance of differentiation at each locus was tested using the log-likelihood (*G*)-based exact test [55] by a MCMC method.

The patterns of spatial genetic structure described as isolation-by-distance (IBD) models [59] were evaluated according to [60] using the Mantel test with 999 random permutations among the matrix of pairwise population differentiation in terms of *F*_ST_/(1 –*F*_ST_) and the matrix of the natural logarithm of geographic distance. These IBD evaluations were tested for all populations and for populations within each island (Amami-Oshima and Tokunoshima) using genalex ver. 6.1 [54].

We applied two methods to assess the populations’ genetic structures. First, we used a Bayesian clustering approach implemented in STRUCTURE ver. 2. 2 [61] to investigate how the genetic variation was organized based on the nSSR data without using any prior information on the populations’ origins. To quantify the amount of variation in the likelihood for each *K* value (i.e. the number of clusters), we performed a series of 20 independent runs for each *K* between 1 and 12. We assumed an admixture model with correlated allele frequencies using a burn-in length of 200,000 and 200,000 MCMC iterations. The programs CLUMPP [62] and DISTRUCT [63] and the *ΔK* statistic [64] were used to determine the appropriate number of clusters (*K*) and to establish the optimal clustering picture for the 20 replicates. These analyses were conducted based on the nine core loci for all populations, and for the full set of 12 loci for populations without *A*. *lutchuense*.

Second, to visualize genetic correlations between each population in the data set, we reconstructed a phylogenetic network (Population Graph)[65] with Genetic Studio [66]. We used all sampling localities as nodes, estimated graphs of the connectivity between the nodes, and performed statistical tests of this connectivity. The resulting network topology was used to identify populations that may have previously experienced or currently be experiencing gene flow. These analyses were performed based on either the nine core loci or the full set of twelve as discussed above.

### Estimation of recent gene flow among species and populations

To estimate the amount and direction of recent (i.e., over the past few generations) gene flow, we followed the method as implemented in BayesAss ver. 1.3 [67]. This method estimates recent migration rates between all pairs of populations, allele frequencies, and inbreeding coefficients for each population. The analyses were performed by identifying individuals as immigrants or descendants of recent immigrants based on the observed temporary disequilibrium of multi-locus genotypic frequencies. Selected parameters (migration rate, allele frequencies, and inbreeding coefficient) were estimated numerically using a MCMC simulation by inferring the corresponding estimated posterior probabilities. To estimate the posterior probability distributions of the parameters, the program was run with a burn-in period of 999,999 interactions and 3 × 10^6^ total interactions. Five independent runs were conducted, and the mean values for each population were compared. Using the results of the genetic clustering analysis as a reference, we analyzed each population from the Amami-Oshima and Tokunoshima islands using the data for the nine core loci.

### Estimation of genetic bottlenecks

To detect recent bottlenecks caused by reductions in effective population size, the observed gene diversity was compared to the equilibrium gene diversity given the observed number of alleles [68, 69] using Bottleneck ver. 1.2.02 [70]. Two models for locus evolution—the infinite allele model (IAM)[71] and stepwise mutation model (SMM)[72]—were used for these analyses, in conjunction with the sign test [72] and the Bayesian Wilcoxon signed-rank test [73]. All of these analyses were performed using 2000 iterations of the coalescent process based on data for the nine core loci in each species.

## Results

### ITS phylogeny

**Fig 3:**
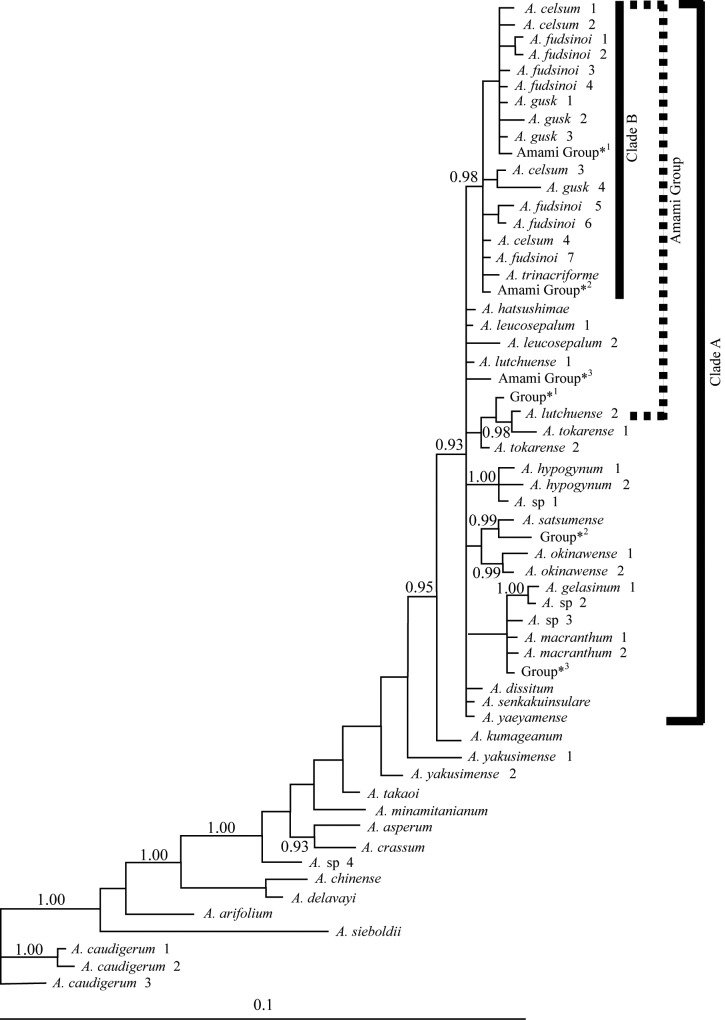
Phylogenetic tree of *Asarum* generated from a Bayesian analysis of the ITS region. BPP values in excess of 90% are highlighted. The taxon labels (including Group*1 and Amami Group*1 –*3) correspond to those used in Table 1.

All sequences have been deposited in DDBJ/GenBank/EMBL: AB699777 –AB699866 (S2 Table). The phylogenetic tree obtained by BI is shown in Fig 3. The nine Amami Group endemics were placed in a large polytomous clade that comprised insular endemics of the Ryukyu Islands, Taiwan, and mainland Japan (Clade A: 0.93 BPP in BI). Furthermore, a robust group that comprised the insular endemics, Clade B, was nested within Clade A (0.98 BPP). The ITS sequence identity among the Clade A samples was 97.7%– 99.8% (668 bp, including indels as differentiation). Most of the nine endemics to the Amami Group possessed several ITS genotypes: nine were identified in *A*. *fudsinoi* (*A*. *fudsinoi* 1–7, Amami Group*1 and Amami Group*2 within Clade B in Fig 3; also see Table 1), six in *A*. *gusk*, five in *A*. *celsum*, three in *A*. *hatsushimae* and *A*. *lutchuense*, two in *A*. *simile* and *A*. *leucosepalum*.

Among the remainder, 25 ITS genotypes from seven of the eight species (*A*. *fudsinoi*, *A*. *celsum*, *A*. *gusk*, *A*. *pellucidum*, and *A*. *trinacriforme* distributed on Amami-Oshima and *A*. *hatsushimae* and *A*. *simile* distributed on Tokunoshima) were assigned to the robust Clade B (0.98 BPP in BI). However, each of these species shared several ITS genotypes and no taxonomic consequence was recognized. The degree of ITS sequence homology among Clade B samples was 98.8%– 99.8%. *A*. *leucosepalum* on Tokunoshima Island had one genotype and formed a polytomy within Clade A.

### Genetic diversity

Table 2 shows the genetic diversity estimates for the studied nSSRs. In total, 196 alleles were detected among all of the studied individuals. The nSSR genetic diversities observed in the 32 studied populations differed between populations, as indicated by the corresponding gene diversity (*H*_S_) values, which ranged from 0.450 to 0.792 with a mean of 0.713. *H*_O_ was relatively low compared to *H*_S_, ranging from 0.267 to 0.618 (mean 0.493). Consequently, the populations’ inbreeding coefficients (*F*_IS_) ranged from 0.157 to 0.531 (mean 0.311). In general, each species had a high level of genetic diversity (*H*_S_), ranging from 0.625 (*A*. *lutchuense*) to 0.777 (*A*. *gusk*). *A*. *lutchuense* had a significantly lower level of genetic diversity (its mean *H*_S_ and *H*_O_ values were 0.625 and 0.360, respectively) and a higher inbreeding coefficient (*F*_IS_ = 0.421) than the other species examined (*P* < 0.05), as well as a significantly higher allelic richness (*AR* = 7.6–8.0). Departures from HWE were observed in all populations (*P* < 0.01).

**Table 2:** Estimation of genetic diversity in 32 populations of nine Asarum species.

Species	Population	N	AR	PA	*H*_O_	*H*_S_	*F*_IS_	*P*-value
*Asarum lutchuense*	Lu1	32	3.468	0.111	0.342	0.634	0.460	<0.01
	Lu2	36	4.089	0.222	0.364	0.695	0.476	<0.01
	Lu3	30	3.742	0	0.344	0.647	0.468	<0.01
	Lu4	10	2.913	0	0.267	0.569	0.531	<0.01
	Lu5	19	3.835	0.444	0.404	0.674	0.401	<0.01
	Lu6	31	4.115	0.111	0.484	0.705	0.314	<0.01
	Lu7	39	2.752	0.444	0.316	0.450	0.297	<0.01
Average		28	3.559	0.190	0.360	0.625	0.421	
*A*. *fudsinoi*	Fu1	11	4.198	0	0.515	0.737	0.301	<0.01
	Fu2	31	4.625	0.111	0.581	0.760	0.236	<0.01
	Fu3	30	4.908	0	0.437	0.750	0.417	<0.01
	Fu4	32	4.696	0.111	0.500	0.744	0.328	<0.01
	Fu5	32	4.774	0.111	0.496	0.748	0.336	<0.01
	Fu6	32	4.863	0	0.518	0.751	0.310	<0.01
Average		28	4.677	0.056	0.508	0.748	0.321	
*A*. *celsum*	Ce1	18	4.498	0.556	0.463	0.747	0.380	<0.01
	Ce2	29	4.963	0	0.525	0.766	0.315	<0.01
	Ce3	21	5.040	0.111	0.513	0.792	0.352	<0.01
	Ce4	16	4.723	0	0.528	0.765	0.310	<0.01
Average		21	4.806	0.167	0.507	0.767	0.339	
*A*. *gusk*	Gu1	24	5.019	0	0.588	0.785	0.251	<0.01
	Gu2	20	4.863	0	0.566	0.770	0.264	<0.01
Average		22	4.941	0	0.577	0.777	0.273	
*A*. *pellucidum*	Pe1	5	4.222	0.111	0.573	0.733	0.218	<0.01
*A*. *trinacriforme*	Tr1	29	4.283	0.111	0.414	0.685	0.396	<0.01
	Tr2	30	5.168	0.222	0.596	0.785	0.241	<0.01
	Tr3	33	4.395	0.000	0.461	0.697	0.339	<0.01
	Tr4	19	4.623	0.222	0.491	0.739	0.335	<0.01
Average		28	4.617	0.139	0.490	0.727	0.328	
*A*. *hatsushimae*	Ha1	29	4.380	0	0.523	0.667	0.216	<0.01
	Ha2	23	4.421	0	0.503	0.680	0.261	<0.01
	Ha3	32	4.735	0	0.549	0.711	0.228	<0.01
Average		28	4.512	0	0.525	0.686	0.235	
*A*. *leucosepalum*	Le1	17	4.933	0.222	0.582	0.762	0.236	<0.01
	Le2	32	4.962	0.556	0.618	0.758	0.185	<0.01
	Le3	32	4.245	0.222	0.569	0.708	0.196	<0.01
Average		27	4.713	0.333	0.590	0.743	0.206	
*A*. *simile*	Si1	32	4.630	0.111	0.551	0.694	0.206	<0.01
	Si2	32	4.599	0	0.607	0.721	0.157	<0.01
Average		32	4.615	0.056	0.579	0.707	0.182	

N: number of samples; AR: allelic richness; PA: private allele; P-value: Hardy-Weinberg equilibrium

### Population genetic structure

**Fig 4:**
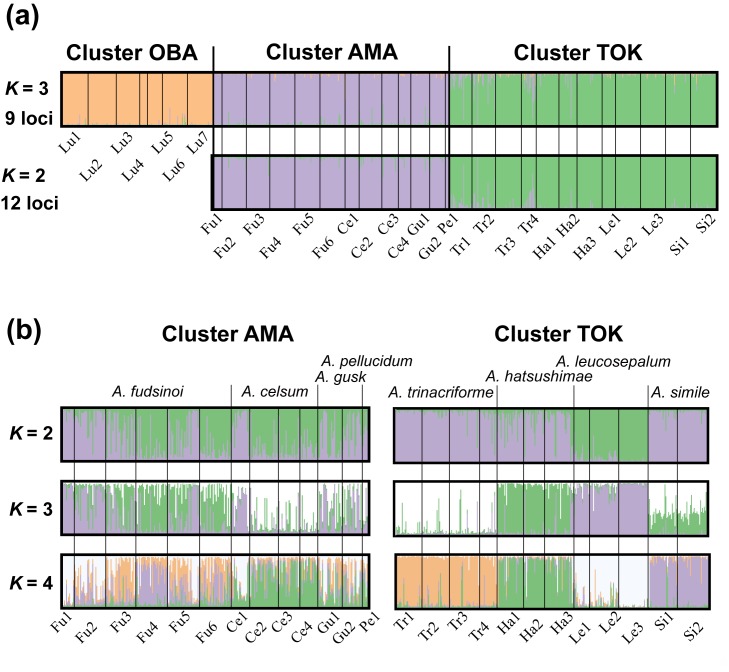
The results of Bayesian clustering analysis using the program STRUCTURE. (a) distribution of cluster membership. Top: analysis of all populations based on the nine core loci with *K* = 3, bottom: analysis of all populations other than those of *A*. *lutchuense* based on 12 loci with *K* = 2; (b) Distribution of AMA and TOK cluster memberships for *K* values of 2–4.

Cluster analysis performed using STRUCTURE based on the nine core loci revealed the existence of three genetically differentiated clusters: OBA, AMA, and TOK (Fig 4A). The clustering patterns for *K* = 3 were highly consistent over 20 independent runs (S2A Fig). Cluster OBA includes all *A*. *lutchuense* populations. Cluster AMA consists of four of the five endemics to Amami-Oshima (*A*. *fudsinoi*, *A*. *celsum*, *A*. *gusk*, and *A*. *pellucidum*), whereas TOK contains all of the endemics to Tokunoshima Island (*A*. *hatsushimae*, *A*. *leucosepalum*, and *A*. *simile*) and one endemic to Amami-Oshima (*A*. *trinacriforme*). Some individuals of *A*. *lutchuense* were admixtures, but most were assigned to a single cluster, OBA (Fig 4A). Thus, although *A*. *lutchuense* occurs frequently with other species on Amami-Oshima, little evidence of hybridization was detected. This clustering pattern agrees well with the ITS phylogeny results. Further analyses of the AMA and TOK clusters were therefore performed using the full set of twelve loci in order to more finely delineate the clustering within them. The results of this analysis were consistent with the original with respect to the clustering of AMA and TOK (*K* = 2), implying that these clusters are robust (Fig 4A).

The AMA and TOK clusters were then analyzed separately (Fig 4B). The clustering patterns within AMA did not correspond to any of the studied species either when using the ‘correct’ number of clusters (*K* = 3) or any other tested cluster number (*K* = 2 or *K* = 4) (S2B Fig). In contrast, the clustering patterns within TOK were such that each population of *A*. *leucosepalum* formed a distinct unit when using the appropriate number of clusters (*K* = 2). Each species unit was also clearly separated at *K* = 4. However, this number of clusters was not supported by either the *ΔK* statistic or CLUMPP analysis.

**Fig 5:**
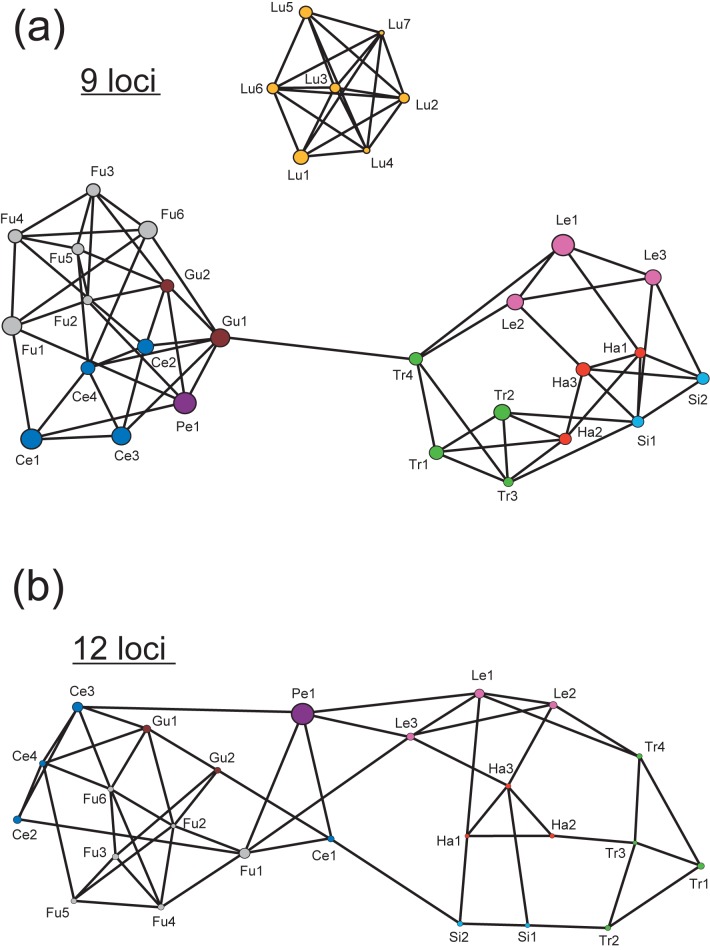
The results of analyses using the POPULATION GRAPH program. (a) all populations based on the nine core loci: (b) eight species (i.e. excluding data for *A*. *lutchuense*) based on 12 loci. Individual species are denoted by circles of different colors: yellow for *A*. *lutchuense* (Lu), blue for *A*. *celsum* (Ce), gray for *A*. *fudsinoi* (Fu), brown for *A*. *gusk* (Gu), dark purple for *A pellucidum* (Pe), green for *A*. *trinacriforme* (Tr), red for *A*. *hatsushimae*, magenta for *A*. *leucosepalum*, and light blue for *A*. *simile*.

A population graph was created that suggested the same genetic correlations as the STRUCTURE analyses (Fig 5). Analysis of the graph (Fig 5A) revealed three distinct genetic units: OBA (seven nodes, 20 edges), AMA (13 nodes, 33 edges), and TOK (12 nodes, 27 edges). The OBA cluster was recognized as an isolated group without any connection to AMA or TOK. AMA was connected to TOK at only one edge, between populations Tr4 and Gu1. This suggests an absence of gene flow between the three clusters, even though sympatric stands of all three clusters’ demes were found on Amami-Oshima. To assess the precise genetic connection between populations, we removed the results for OBA from the dataset and repeated the analysis using data for 12 loci (Fig 5B). This second analysis supported the robustness of the AMA (13 nodes, 29 edges) and TOK (12 nodes, 22 edges) clusters. There were four connecting edges between the AMA and TOK clusters; the probability that this occurred by chance was very low (7.7 × 10^−6^). Due to the small size of the Pe1 stand, which consisted of only five individuals, a re-analysis was performed in which the Pe1 data were excluded. This yielded the same topology as the preceding analysis (data not shown). It is noteworthy that the sympatric stands on Amami-Oshima (e.g., Fu3 and Ce2; Fu4, Ce4, Pe1, and Tr2) were not connected in the population graph but some allopatric stands of different species and/or which were isolated by long distances were connected (e.g., Fu1 and Pe1; Si1 and Tr2) (Fig 5B).

### Gene flow within sympatric stands across species and stands within a species

**Fig 6:**
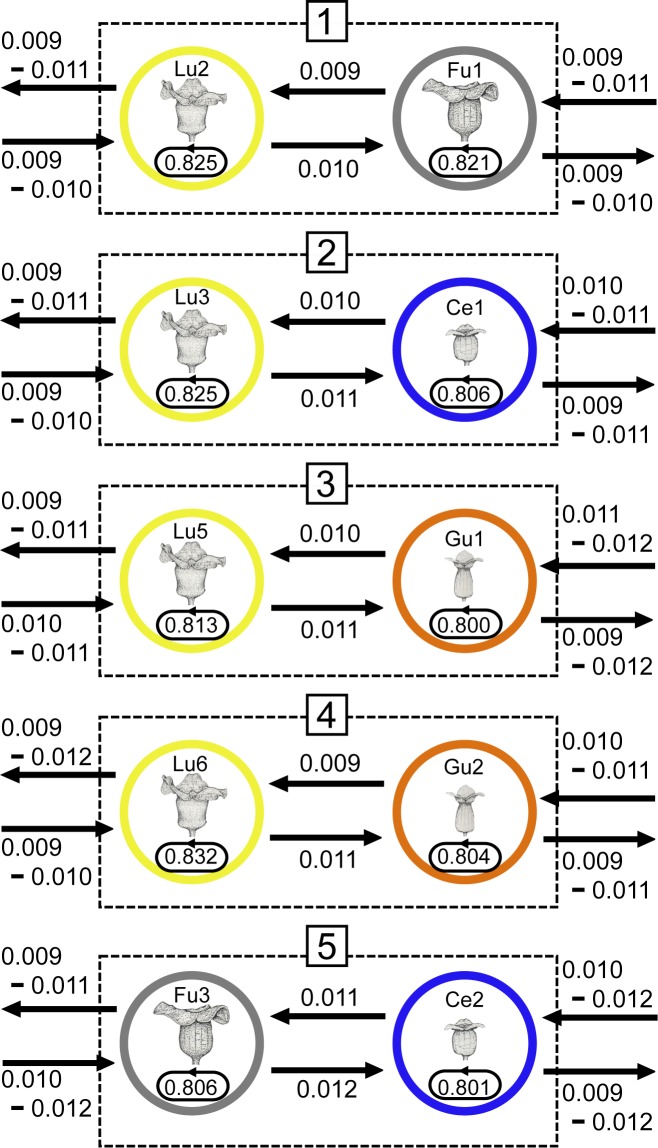
Mean values for the posterior distribution of the migration rate (*m*) of each *Asarum* populations within “sympatric stands” on Amami Oshima estimated using BayesAss. Estimated migration rates are shown between species in “sympatric stands” 1–5, each of which contains two species. The estimates for “sympatric stands” 1 – 4 were based on data for the nine core loci while those for “sympatric stands” 5 were based on data for the full set of 12 loci. The stand and population abbreviations correspond to those used in Fig 1, S3 Table, S4 Table and S5 Table.

The degree of gene flow was evaluated using BayesAss 1.3 for “sympatric stands” on Amami-Oshima (stands 1–6 in Fig 1B) and for allopatric stands. In sympatric stands 1–4, which contain *A*. *lutchuense* along with some other species [*A*. *fudsinoi* (pop 1), *A*. *celsum* (pop 2), and *A*. *gusk* (pops 3, 4)], most gene flow occurred within the same species and very little gene flow occurred across species based on data for the nine core loci. For example, in sympatric stand 1, the migration rate (mean value) within *A*. *lutchuense* (from Lu2 to Lu2) was 0.825, whereas the rates from *A*. *lutchuense* (Lu2) to *A*. *fudsinoi* (Fu1) and in the reverse direction (from Fu1 to Lu2) were 0.010 and 0.009, respectively (Fig 6; S3 Table).

**Fig 7:**
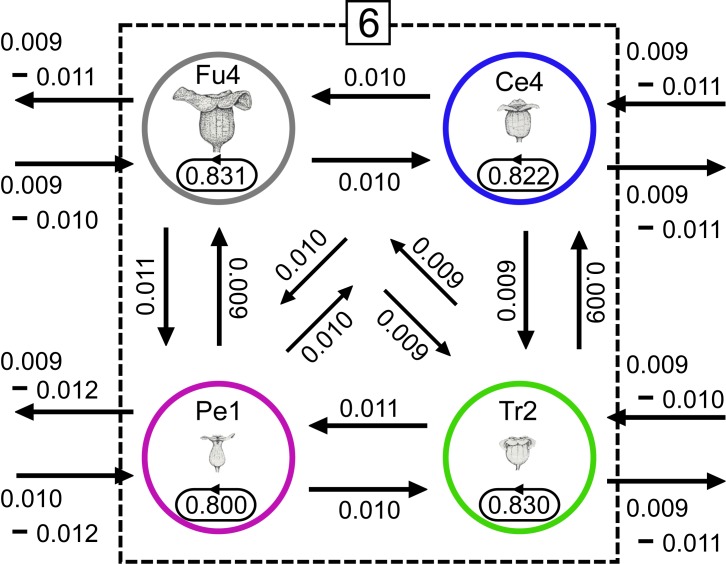
Mean values for the posterior distribution of the migration rate (*m*) of each *Asarum* population in sympatric stand 6, in which four species grow sympatrically on Amami Oshima. Estimated migration rates were based on data for the full set of 12 loci. The stand and population abbreviations correspond to those used in Fig 1, S3 Table, S4 Table and S5 Table.

Because the degree of genetic differentiation among populations was typically greater than 0.05 (*F*_ST_ ≥ 0.05), reliable estimates of migration rates could be made. The rates of migration between species for sympatric stands 5 (Fig 6) and 6 (Fig 7) were estimated to be low based on data for 12 loci. For example, sympatric stand 6 (Fig 7) contains four species (*A*. *fudsinoi*, *A*. *celsum*, *A*. *pellucidum*, and *A*. *trinacriforme*). Most of the gene flow in this stand occurred within individual species, however, and that between species was comparatively limited.

The migration rates between allopatric stands within individual species were very low, suggesting restricted gene flow between stands. Allopatric stands Ce2 and Ce3 (*A*. *celsum*) were located in close proximity to one-another (*c*. 700 m), but their migration rates were low (*m* = 0.011) (S3 Table).

### Genetic differentiation among species and stands

The degree of genetic differentiation among the nine species, represented by their pairwise *F*_ST_ values, was in good agreement with the results of STRUCTURE analysis: genetic differentiation was high among the OBA, AMA, and TOK clusters, but low among species within the same cluster. Pairwise *F*_ST_ values were also determined between the OBA cluster, which consists exclusively of *A*. *lutchuense*, and the other species based on the nine core loci (Table 3, upper diagonal). The mean *F*_ST_ value for OBA and members of the AMA cluster was *c*. 0.21 based on individual *F*_ST_ values of 0.185–0.266 while that for OBA and members of the TOK cluster was *c*. 0.23 based on a range of 0.21–0.266. In contrast, the pairwise *F*_ST_ values between species belonging to the AMA and TOK clusters were lower (*c*. 0.126, *F*_ST_ = 0.096–0.193), based on the full set of 12 loci (Table 3, lower diagonal). Thus, *A*. *lutchuense* aside, the endemic species of the Amami group exhibit a high degree of homogeneity.

**Table 3:** Pairwise FST comparisons between nine Asarum species on Amami Group.

	*A*. *fudsinoi*	*A*. *celsum*	*A*. *gusk*	*A*. *pellucidum*	*A*. *trinacriforme*	*A*. *hatsushimae*	*A*. *leucosepalum*	*A*. *simile*
*A*. *lutchuense*	**0.193**	**0.188**	**0.185**	**0.255**	**0.211**	**0.266**	**0.213**	**0.261**
*A*. *fudsinoi*		0.028	0.016	0.065	**0.125**	**0.140**	**0.136**	**0.147**
*A*. *celsum*	0.025		0.019	0.085	**0.116**	**0.145**	**0.129**	**0.138**
*A*. *gusk*	0.015	0.019		0.071	**0.115**	**0.142**	**0.125**	**0.140**
*A*. *pellucidum*	0.056	0.076	0.067		**0.168**	**0.226**	**0.191**	**0.223**
*A*. *trinacriforme*	**0.102**	0.096	0.096	**0.147**		0.066	0.062	0.053
*A*. *hatsushimae*	**0.119**	**0.128**	**0.125**	**0.191**	0.055		0.081	0.045
*A*. *leucosepalum*	**0.127**	**0.123**	**0.126**	**0.173**	0.065	0.069		0.095
*A*. *simile*	**0.123**	**0.115**	**0.119**	**0.193**	0.044	0.047	0.094	

Values above and below the diagonals are calculated from nine loci (availabe for all species) and 12 loci (available except A. lutchuense), respectively.

Values above 0.1 are in bold.

The degree of genetic differentiation among populations within individual species was low (S4 Table). For example, the *F*_ST_ values for *A*. *fudsinoi* on Amami-Oshima ranged from 0.01 to 0.08 with a mean of 0.05. Interestingly, similar levels of differentiation were observed between different species within the AMA cluster. For example, the mean *F*_ST_ value between populations of *A*. *fudsinoi* and *A*. *celsum* was 0.06 (*F*_ST_ = 0.03–0.10). These low *F*_ST_ values suggest that there is negligible genetic differentiation between populations of the same species and also between those of different species. The Mantel test did not indicate any IBD among the populations of individual species for any of the nine insular endemics (*P* > 0.05; Table 4).

**Table 4:** Results of Mantel test for the Asarum species on Amami Group.

Species*	Number of populations	Mantel's *R*	*P* value
*Asarum lutchuense*	6	0.001	0.624
*A*. *fudsinoi*	6	0.369	0.057
*A*. *celsum*	4	0.249	0.129
*A*. *trinacriforme*	4	0.210	0.234
*A*. *hatsushimae*	3	0.773	0.174
*A*. *leucosepalum*	3	0.620	0.324

*Species with >3 populations was analyzed.

### Evidence of a bottleneck

The results of tests for mutation–drift equilibrium are presented in S5 Table. Almost all populations showed evidence of a recent bottleneck under both the IAM and the SMM (*P* < 0.05; two-tailed sign and Wilcoxon tests). Under the IAM, 14 populations exhibited a significant heterozygosity excess (i.e. evidence of a recent bottleneck) in both the sign and Wilcoxon tests. Under the SMM, significant bottleneck effects were detected in three populations using the sign and Wilcoxon tests.

## Discussion

The key findings of this investigation of nine *Asarum* insular endemic species on Japan’s small continental islands were that (1) the species were divided into three robust genetic clusters; (2) there was very little genetic differentiation among species within a cluster, indicating incomplete lineage sorting; (3) each species was maintained by outcrossing accompanied by inbreeding within populations; and (4) each species was highly isolated even within sympatric stands—the species were reproductively isolated.

### Maintenance of species cohesion

The main objective of this work was to determine how the nine studied insular *Asarum* species have maintained their species cohesion within the small islands of the Amami Group. Two potential explanations were considered: (i) prezygotic isolation due to a high degree of species differentiation with respect to floral morphology that imposes reproductive isolation, or (ii) a trend towards inbreeding, which could cause reproductive isolation even in sympatric stands.

Flowers of the nine endemics are quite different in size, calyx tube mouth size (throat), color, calyx tube shape, shape of pistil appendage, and pedicel length. For example, calyx tube mouth size would be critical to select pollinator fauna: very narrow throat of *Asarum gusk* and *A*. *pellucidum* would limits pollinators to particular fauna with small body size such as mites or thrips, whereas wide throat of *A*. *fudsinoi* and *A*. *lutchuense* would be able to accept larger ones such as fungus gnat or amphipods inhabiting on forest floor. Most flowers conform to these morphologies as summarized in Fig 2 and S1 Table, suggesting a possibility of pollinator fauna differentiation among the species.

According to the prezygotic isolation hypothesis, the morphological differentiation of each species’ flowers suggests that each *Asarum* species is exposed to selection by species-specific pollinators that are adapted to their unique floral size and/or structure, and that occasional hybrids are removed by pollinator preference selection. This could be taken to imply that the endemics within each cluster originated from a common ancestor over a relatively short period during which few genetic differences accumulated between the species but their floral morphologies diverged rapidly and substantially, causing reproductive isolation as each one adapted to different pollinators. It is well known that species-specific morphologies can be established and sustained by stabilizing selection of this sort [74, 75].

Inbreeding within each species could also cause reproductive isolation in sympatric stands. The high genetic diversity of the nine *Asarum* species (*H*_S_ = 0.625–0.777) suggests that each species is maintained by outcrossing, but the highly positive *F*_IS_ values for each endemic (0.182–0.421) suggest that inbreeding also occurred. Inbreeding may be one of the factors responsible for reproductive isolation in sympatric stands. Although *Asarum* flower is not herkogamy, previous studies have also suggested that *Asarum* can self-pollinate [76. 77], possibility due to self-pollination within a flower caused by ground hovering flower visitors (e.g., terrestrial amphipods, small spiders, and ants: Y. Maeda personal observation) which may continue staying in calyx tube for a long time. A mixed mating system involving both outcrossing and inbreeding could thus be responsible for the tight species cohesion observed even among sympatric stands in the Amami islands.

Thus, we can not judge the contribution of floral differentiation vs. inbreeding to the maintenance of species cohesion, currently. To further clarify the situation, it would be desirable to identify the pollinators of each studied *Asarum* species. Some *Asarum* pollinators such as fungus gnats (Mycetophilidae) have been identified in North America [78] and mainland Japan [79] and the genus’ floral scent emissions have been studied [80]. However, we have not yet finished to identify its pollinators in the Amami Islands.

### Geographical implications for speciation

The genetic structure of the TOK cluster, which consists of three endemics to Tokunoshima and *A*. *trinacriforme* from Amami-Oshima, suggests that speciation of the insular endemics (both *A*. *trinacriforme* and other species) may have occurred across the islands. Because *Asarum* seeds are dispersed by ants and/or gravity, a continuous landmass between the islands must be postulated to explain the genetic structure of TOK and the distribution of *A*. *lutchuense*. Continuous land bridges between Amami-Oshima and Tokunoshima are assumed to have existed on several occasions during the Quaternary (due to glacial regression), with the most recent having formed during the Last Glacial Maximum (LGM), *c*. 20,000 years ago [39, 40].

Within individual islands, there was no evidence of IBD among allopatric stands of individual species. The low observed rates of genetic differentiation and migration rates could be due to gradual genetic differentiation across species, possibly due to rapid adaptive radiation from the common ancestor over a short period of time. The insular endemics differentiated within a short period of time, and their lineage sorting is probably still incomplete.

## Supporting information

S1 Fig Photographs of a sympatric zone habitat. Top: *Asarum lutchuense* (yellow) and *A*. *celsum* (red) at Kinsakubaru, bottom: *A*. *lutchuense* (yellow) and *A*. *gusk* (red) at Mt. Takinohana (stands are numbers 2 and 4 in Fig 1, respectively).
(EPS)

S2 Fig Results of Bayesian clustering analysis using the program STRUCTURE. (a) values of Δ*K* and the similarity coefficient *H* from the data in Fig 4A; (b) values of Δ*K* and the similarity coefficients *H* from the data in Fig 4B.
(EPS)

S1 Table Variability of the morphology of nine *Asarum* species in the Amami Group (after Hatusima & Yamahata 1988). (PDF)

S2 Table Materials used in ITS phylogenetic analysis. (PDF)

S3 Table Estimated migration rates between populations within Amami-Oshima Island. (PDF)

S4 Table Pairwise *F*ST values for the 32 studied *Asarum* populations in the Amami Group. (PDF)

S5 Table Probability of a bottleneck effect. Probabilities of significant (*P* < 0.05) heterozygosity excess for the two-tailed sign and Wicoxon tests under the infinite allele mutation model (IAM) and stepwise mutation model (SMM) are indicated with asterisks.
(PDF)
