# Supplementary material for: Tight species cohesion among sympatric insular wild gingers (Asarum spp. Aristolochiaceae) on continental islands: Highly differentiated floral characteristics versus undifferentiated genotypes
Source: PLoS One. 2017 Mar 16;12(3):e0173489. doi: 10.1371/journal.pone.0173489 (PMC5354281; doi:10.1371/journal.pone.0173489)
Supplement: S1 Table — (PDF) [file pone.0173489.s003.pdf]

S1 Table Variability of morphology of nine *Asarum* species in Amami Group (after Hatusima & Yamahata 1988)

Species	Distribution*	Flower								Leaf blade			Flowering season***
		Shape	Color**	Throat width (mm)	Length (mm)	Width of calyx tube (mm)	Length of peduncle (mm)	No. of carpels and stamens	Trichome	Length (cm)	Width (cm)	Adaxial surface	
A. lutchuense	A, T	tubular	G, R	8.0	12-15	12-15	< 10	6 and 12	hispid	10-20	6-15	lustrous	November to January
A. celsum	A	tubular	G, R	3.1	8-10	10	< 10	6 and 12	hispid	5-8	3-6	lusterless	December to March
A. fudsinoi	A	tubular	G, R	5.3	15-30	15-20	< 10	6 and 12	glabrous	10-22	6-15	lustrous	December to June
A. gusk	A	urceolate	G, R	3.4	11	8-9	< 10	6 and 12	hispid	8-9	4-5	lusterless	January to April
A. pellucidum	A	urceolate	G, R	1.7	8	8	< 10	3 and 6	hispid	6-7	3-4	lusterless	December to January
A. trinacriforme	A	tubular	G, R	2.5	6-9	6-10	< 10	3 and 6	hispid	5-7	3-5	lusterless	March to May
A. hatsushimae	T	tubular	G, R	2.8	15-20	12-15	20-60	6 and 12	hispid	7-12	5-7	lusterless	March to April
A. leucosepalum	T	tubular	WR	3.3	8-12	7-10	20-30	6 and 12	hispid	6-12	3-7	lusterless	March
A. simile	T	tubular	G, R	4.0	10-13	12-15	< 10	6 and 12	hispid	7-12	5-7	lusterless	March to April

* Abbreviations: A, Amami Oshima Island; T, Tokunoshima Island.

**Abbreviations: G, Green; R, Reddish green; WR, Reddish greenwith white layx lobes.

***Based on obsevation between 2003-2010.
