# Supplementary material for: Tight species cohesion among sympatric insular wild gingers (Asarum spp. Aristolochiaceae) on continental islands: Highly differentiated floral characteristics versus undifferentiated genotypes
Source: PLoS One. 2017 Mar 16;12(3):e0173489. doi: 10.1371/journal.pone.0173489 (PMC5354281; doi:10.1371/journal.pone.0173489)
Supplement: S2 Table — (PDF) [file pone.0173489.s004.pdf]

S2 Table Materials used in ITS phylogenetic analysis

Taxon label	Taxon	Distribution	Accession Number
	sect. *Heterotropa*		
	Species from Amami Group		
Asarum celsum 1	*A. celsum*	Amami Oshima Island*	AB699782
A. celsum 2	*A. celsum*	Amami Oshima Island*	AB699783, AB699784
A. celsum 3	*A. celsum*	Amami Oshima Island*	AB699785, AB699786, AB699787
A. celsum 4	*A. celsum*	Amami Oshima Island*	AB699788, AB699789, AB699790
A. fudsinoi 1	*A. fudsinoi*	Amami Oshima Island*	AB699798, AB699799
A. fudsinoi 2	*A. fudsinoi*	Amami Oshima Island*	AB699800, AB699801
A. fudsinoi 3	*A. fudsinoi*	Amami Oshima Island*	AB699802, AB699803
A. fudsinoi 4	*A. fudsinoi*	Amami Oshima Island*	AB699804
A. fudsinoi 5	*A. fudsinoi*	Amami Oshima Island*	AB699805
A. fudsinoi 6	*A. fudsinoi*	Amami Oshima Island*	AB699806
A. fudsinoi 7	*A. fudsinoi*	Amami Oshima Island*	AB699807
A. gusk 1	*A. gusk*	Amami Oshima Island*	AB699817
A. gusk 2	*A. gusk*	Amami Oshima Island*	AB699819
A. gusk 3	*A. gusk*	Amami Oshima Island*	AB699820, AB699821, AB699822
A. gusk 4	*A. gusk*	Amami Oshima Island*	AB699818
A. hatsushimae	*A. hatsushimae*	Tokunoshima Island*	AB699826, AB699827
A. leucosepalum 1	*A. leucosepalum*	Tokunoshima Island*	AB699835
A. leucosepalum 2	*A. leucosepalum*	Tokunoshima Island*	AB699866
A. lutchuense 1	*A. lutchuense*	Tokunoshima Island*	AB699863
A. lutchuense 2	*A. lutchuense*	Amami Oshima Island*	AB699836
A. trinacriforme	*A. trinacriforme*	Amami Oshima Island*	AB699850
Group*1	*A. lutchuense*	Amami Oshima Island, Tokunoshima Island*	AB699837, AB699838
Amami Group*1	*A. fudsinoi*	Amami Oshima Island*	AB699808, AB699809
	A. simile	Tokunoshima Island*	AB699854
Amami Group*2	*A. celsum*	Amami Oshima Island*	AB699791, AB699792, AB699793
	A. fudsinoi	Amami Oshima Island*	AB699810, AB699811, AB699812
	A. gusk	Amami Oshima Island*	AB699823, AB699824, AB699825
	A. pellucidum	Amami Oshima Island*	AB700595
	A. hatsushimae	Tokunoshima Island*	AB699828, AB699829, AB699830
	A. simile	Tokunoshima Island*	AB699855, AB699856, AB699857
Amami Group*3	*A. gusk*	Amami Oshima Island*	AB700594
	A. hatsushimae	Tokunoshima Island*	AB699831, AB699832

S3 Table (Continued)

Taxon label	Taxon	Distribution	Accession Number
Species outsude of Amami Group			
A. asperum	*A. asperum*	Mainland Japan	AB699781
A. hypogynum	*A. hypogynum*	Taiwan	AB699833
A. hypogynum 2	*A. hypogynum*	Taiwan	AB699861
A. kumageanum	*A. kumageanum*	Yakushima Island*	AB699834
A. macranthum 1	*A. macranthum*	Taiwan	AB699840
A. macranthum 2	*A. macranthum*	Taiwan	AB699860
A. minamitanianum	*A. minamitanianum*	Mainland Japan	AB699841
A. okinawense 1	*A. okinawense*	Okinawajima Island*	AB699842
A. okinawense 2	*A. okinawense*	Okinawajima Island*	AB699843
A. satsumense	*A. satsumense*	Mainland Japan	AB699844
A. senkakuinsulare	*A. senkakuinsulare*	Uotsuri Island*	AB699845
A. chinense	*A. chinense*	Central China	AB699794
A. crassum	*A. crassum*	Iejima Island*	AB699795
A. delavayi	*A. delavayi*	Central China	AB699796
A. dissitum	*A. dissitum*	Ishigakijima Island*	AB699797
A. gelasinum	*A. gelasinum*	Iriomotejima Island*	AB699813, AB699814
A. sp 1	*A.* sp	Taiwan	AB699859
A. sp 2	*A.* sp	Okinawajima Island*	AB699816
A. sp 3	*A.* sp	Okinawajima Island*	AB699815
A. sp 4	*A.* sp	Kuroshima Island*	AB699862
A. takaoi	*A. takaoi*	Mainland Japan	AB699849
A. tokarense 1	*A. tokarense*	Kuchinoshima Island*	AB699864
A. tokarense 2	*A. tokarense*	Nakanoshima Island*	AB699865
A. yaeyamense	*A. yaeyamense*	Iriomotejima Island*	AB699851
A. yakusimense 1	*A. yakusimense*	Yakushima Island*	AB699852
A. yakusimense 2	*A. yakusimense*	Yakushima Island*	AB699853
	A. tokarense	Nakanoshima Island*	AB699839
Group*2	*A. asaroides*	Mainland Japan	AB699846
	A. unzen	Mainland Japan	AB699847
Group*3	*A. monodoriflorum*	Iriomotejima Island*	AB699848
	A. albomaculata	Taiwan	AB699859
Outgroups			
	**sect. *Asarum***		
A. caudigerum 1	*Asarum caudigerum*	Okinawajima Island*	AB699777
A. caudigerum 2	*A. caudigerum*	Okinawajima Island*	AB699778
A. caudigerum 3	*A. caudigerum*	Taiwan	AB699779
	**sect. *Asiasarum***		
A. sieboldii	*A. sieboldii*	Mainland Japan, Korean Peninsula, China	AB699780
	**sect. *Hexastylis***		
A. arifolium	*A. arifolium*	USA	AB699858

* Islands of Ryukyu Archipelago
