# Supplementary material for: Tight species cohesion among sympatric insular wild gingers (Asarum spp. Aristolochiaceae) on continental islands: Highly differentiated floral characteristics versus undifferentiated genotypes
Source: PLoS One. 2017 Mar 16;12(3):e0173489. doi: 10.1371/journal.pone.0173489 (PMC5354281; doi:10.1371/journal.pone.0173489)
Supplement: S3 Table — (PDF) [file pone.0173489.s005.pdf]

S3 Table Estimated migration rates between populations within Amami-Oshima Island.

To	From																		
	Lu2	Lu3	Lu5	Lu6	Fu1	Fu2	Fu3	Fu4	Fu5	Ce1	Ce2	Ce3	Ce4	Gu1	Gu2	Pe1	Tr1	Tr2	Tr3
Lu2	**0.825**	0.010	0.010	0.009	0.009	0.010	0.010	0.009	0.010	0.010	0.010	0.009	0.010	0.010	0.010	0.010	0.010	0.010	0.010
Lu3	0.010	**0.825**	0.010	0.010	0.010	0.010	0.009	0.009	0.009	0.010	0.010	0.010	0.009	0.010	0.010	0.010	0.010	0.009	0.010
Lu5	0.010	0.010	**0.813**	0.011	0.010	0.010	0.010	0.011	0.010	0.011	0.010	0.010	0.010	0.010	0.011	0.011	0.010	0.011	0.011
Lu6	0.009	0.009	0.009	**0.832**	0.009	0.009	0.009	0.009	0.009	0.010	0.009	0.009	0.009	0.009	0.009	0.009	0.009	0.009	0.010
Fu1	0.010	0.010	0.010	0.010	**0.821**	0.010	0.009	0.009	0.010	0.010	0.010	0.010	0.010	0.010	0.010	0.011	0.010	0.010	0.010
Fu2	0.010	0.011	0.011	0.010	0.011	**0.804**	0.010	0.011	0.011	0.011	0.012	0.011	0.010	0.011	0.011	0.012	0.011	0.010	0.011
Fu3	0.010	0.011	0.011	0.012	0.011	0.011	**0.806**	0.011	0.010	0.011	0.011	0.011	0.010	0.010	0.011	0.011	0.012	0.011	0.010
Fu4	0.010	0.009	0.009	0.009	0.009	0.009	0.010	**0.831**	0.009	0.010	0.009	0.010	0.010	0.010	0.010	0.009	0.009	0.009	0.010
Fu5	0.011	0.011	0.011	0.011	0.011	0.011	0.010	0.011	**0.804**	0.011	0.011	0.010	0.011	0.011	0.011	0.011	0.012	0.011	0.011
Ce1	0.010	0.011	0.011	0.010	0.010	0.011	0.010	0.011	0.011	**0.806**	0.011	0.011	0.011	0.011	0.011	0.011	0.011	0.011	0.010
Ce2	0.011	0.011	0.011	0.010	0.011	0.012	0.012	0.011	0.011	0.011	**0.801**	0.011	0.011	0.010	0.011	0.012	0.011	0.010	0.011
Ce3	0.011	0.011	0.010	0.010	0.011	0.011	0.011	0.010	0.011	0.011	0.011	**0.809**	0.010	0.011	0.010	0.012	0.011	0.010	0.010
Ce4	0.010	0.010	0.010	0.010	0.010	0.010	0.010	0.010	0.010	0.010	0.011	0.009	**0.822**	0.010	0.009	0.010	0.010	0.010	0.010
Gu1	0.011	0.011	0.011	0.011	0.011	0.012	0.011	0.011	0.011	0.011	0.011	0.012	0.011	**0.800**	0.011	0.011	0.012	0.011	0.011
Gu2	0.010	0.011	0.011	0.011	0.011	0.011	0.011	0.011	0.011	0.011	0.011	0.011	0.011	0.011	**0.804**	0.011	0.011	0.010	0.011
Pe1	0.011	0.011	0.011	0.011	0.011	0.011	0.011	0.011	0.011	0.011	0.012	0.011	0.010	0.012	0.011	**0.800**	0.012	0.011	0.011
Tr1	0.010	0.009	0.010	0.010	0.010	0.010	0.010	0.010	0.010	0.010	0.009	0.009	0.010	0.009	0.010	0.011	**0.826**	0.010	0.010
Tr2	0.009	0.009	0.010	0.009	0.010	0.009	0.010	0.009	0.009	0.009	0.010	0.010	0.009	0.010	0.009	0.010	0.009	**0.830**	0.010
Tr3	0.010	0.011	0.011	0.011	0.010	0.010	0.011	0.011	0.010	0.011	0.011	0.011	0.011	0.011	0.011	0.010	0.011	0.010	**0.809**

Non-migration rate in each population is marked in bold.
