# Supplementary material for: Tight species cohesion among sympatric insular wild gingers (Asarum spp. Aristolochiaceae) on continental islands: Highly differentiated floral characteristics versus undifferentiated genotypes
Source: PLoS One. 2017 Mar 16;12(3):e0173489. doi: 10.1371/journal.pone.0173489 (PMC5354281; doi:10.1371/journal.pone.0173489)
Supplement: S4 Table — (PDF) [file pone.0173489.s006.pdf]

S4 Table Pairwise F_{ST} values for the 32 studied *Asarum* populations in the Amami Group.

	Lu1	Lu2	Lu3	Lu4	Lu5	Lu6	Lu7	Fu1	Fu2	Fu3	Fu4	Fu5	Fu6	Ce1	Ce2	Ce3	Ce4	Gu1	Gu2	Pe1	Tr1	Tr2	Tr3	Tr4	Ha1	Ha2	Ha3	Le1	Le2	Le3	Si1	Si2
Lu1		0.04	0.08	0.07	0.08	0.06	**0.22**	**0.27**	**0.25**	**0.24**	**0.25**	**0.23**	**0.24**	**0.26**	**0.22**	**0.23**	**0.22**	**0.22**	**0.22**	**0.30**	**0.30**	**0.22**	**0.27**	**0.25**	**0.31**	**0.30**	**0.29**	**0.25**	**0.24**	**0.26**	**0.30**	**0.28**
Lu2			0.04	0.04	0.03	0.05	**0.17**	**0.23**	**0.21**	**0.21**	**0.21**	**0.20**	**0.21**	**0.22**	**0.19**	**0.20**	**0.18**	**0.18**	**0.19**	**0.24**	**0.26**	**0.19**	**0.23**	**0.21**	**0.27**	**0.27**	**0.25**	**0.20**	**0.21**	**0.23**	**0.26**	**0.24**
Lu3				0.08	0.06	0.04	**0.16**	**0.25**	**0.23**	**0.24**	**0.23**	**0.23**	**0.22**	**0.25**	**0.21**	**0.22**	**0.22**	**0.21**	**0.21**	**0.27**	**0.28**	**0.22**	**0.26**	**0.24**	**0.30**	**0.30**	**0.28**	**0.24**	**0.24**	**0.26**	**0.28**	**0.26**
Lu4					0.08	0.07	**0.26**	**0.28**	**0.27**	**0.27**	**0.28**	**0.26**	**0.26**	**0.28**	**0.24**	**0.25**	**0.24**	**0.23**	**0.25**	**0.31**	**0.30**	**0.22**	**0.28**	**0.25**	**0.32**	**0.32**	**0.30**	**0.27**	**0.26**	**0.29**	**0.30**	**0.28**
Lu5						0.06	**0.21**	**0.25**	**0.22**	**0.22**	**0.22**	**0.22**	**0.22**	**0.23**	**0.19**	**0.20**	**0.19**	**0.19**	**0.20**	**0.26**	**0.28**	**0.20**	**0.25**	**0.23**	**0.29**	**0.28**	**0.27**	**0.21**	**0.22**	**0.24**	**0.27**	**0.26**
Lu6							**0.18**	**0.23**	**0.21**	**0.20**	**0.21**	**0.19**	**0.19**	**0.21**	**0.17**	**0.18**	**0.17**	**0.18**	**0.18**	**0.23**	**0.26**	**0.19**	**0.24**	**0.21**	**0.27**	**0.26**	**0.25**	**0.20**	**0.21**	**0.23**	**0.26**	**0.25**
Lu7								**0.39**	**0.34**	**0.32**	**0.33**	**0.32**	**0.33**	**0.36**	**0.31**	**0.33**	**0.33**	**0.32**	**0.32**	**0.44**	**0.40**	**0.33**	**0.36**	**0.38**	**0.40**	**0.40**	**0.38**	**0.35**	**0.34**	**0.37**	**0.40**	**0.40**
Fu1									0.05	0.09	**0.10**	0.09	0.07	**0.12**	**0.10**	0.09	**0.10**	0.08	0.08	0.06	**0.20**	**0.16**	**0.21**	**0.18**	**0.22**	**0.22**	**0.20**	**0.17**	**0.19**	**0.21**	**0.21**	**0.20**
Fu2								0.04		0.05	0.07	0.04	0.06	0.08	0.07	0.06	0.07	0.06	0.03	0.08	**0.17**	**0.13**	**0.19**	**0.16**	**0.18**	**0.17**	**0.15**	**0.16**	**0.16**	**0.20**	**0.17**	**0.16**
Fu3								0.08	0.04		0.04	0.01	0.05	0.08	0.05	0.05	0.04	0.06	0.02	0.09	**0.19**	**0.13**	**0.20**	**0.16**	**0.17**	**0.15**	**0.15**	**0.15**	**0.15**	**0.20**	**0.18**	**0.17**
Fu4								0.08	0.05	0.04		0.05	0.05	0.09	0.05	0.05	0.05	0.06	0.04	**0.10**	**0.19**	**0.13**	**0.21**	**0.17**	**0.19**	**0.18**	**0.17**	**0.16**	**0.16**	**0.19**	**0.20**	**0.18**
Fu5								0.08	0.03	0.01	0.04		0.05	0.08	0.05	0.05	0.03	0.04	0.02	0.09	**0.18**	**0.12**	**0.18**	**0.15**	**0.15**	**0.14**	**0.13**	**0.15**	**0.15**	**0.18**	**0.15**	**0.14**
Fu6								0.06	0.05	0.05	0.05	0.05		**0.11**	0.05	0.05	0.04	0.04	0.06	**0.10**	**0.17**	**0.11**	**0.19**	**0.16**	**0.16**	**0.17**	**0.16**	**0.15**	**0.15**	**0.19**	**0.16**	**0.15**
Ce1								**0.10**	0.08	0.08	0.08	0.07	**0.10**		0.07	0.07	0.05	0.07	0.06	**0.12**	**0.18**	**0.12**	**0.20**	**0.17**	**0.18**	**0.16**	**0.16**	**0.16**	**0.16**	**0.19**	**0.17**	**0.16**
Ce2								0.08	0.06	0.05	0.04	0.04	0.05	0.06		0.01	0.02	0.04	0.04	**0.11**	**0.17**	**0.09**	**0.18**	**0.17**	**0.18**	**0.17**	**0.16**	**0.15**	**0.15**	**0.17**	**0.17**	**0.15**
Ce3								0.08	0.06	0.04	0.05	0.05	0.04	0.07	0.01		0.02	0.02	0.03	0.08	**0.17**	**0.10**	**0.17**	**0.15**	**0.16**	**0.15**	**0.15**	**0.14**	**0.14**	**0.17**	**0.15**	**0.15**
Ce4								0.08	0.06	0.04	0.05	0.03	0.04	0.05	0.02	0.01		0.03	0.04	**0.10**	**0.17**	**0.09**	**0.17**	**0.15**	**0.15**	**0.14**	**0.14**	**0.14**	**0.14**	**0.16**	**0.15**	**0.13**
Gu1								0.07	0.05	0.05	0.06	0.03	0.04	0.07	0.04	0.03	0.03		0.03	**0.10**	**0.16**	**0.09**	**0.17**	**0.12**	**0.16**	**0.15**	**0.14**	**0.13**	**0.13**	**0.15**	**0.14**	**0.13**
Gu2								0.07	0.03	0.02	0.04	0.01	0.05	0.06	0.03	0.02	0.04	0.02		0.06	**0.18**	**0.12**	**0.18**	**0.14**	**0.16**	**0.15**	**0.14**	**0.15**	**0.14**	**0.18**	**0.17**	**0.16**
Pe1								0.06	0.07	0.08	0.09	0.08	0.09	**0.12**	0.09	0.08	0.09	0.09	0.05		**0.22**	**0.17**	**0.22**	**0.16**	**0.24**	**0.23**	**0.21**	**0.20**	**0.19**	**0.23**	**0.23**	**0.22**
Tr1								**0.16**	**0.14**	**0.16**	**0.16**	**0.15**	**0.15**	**0.15**	**0.14**	**0.14**	**0.14**	**0.13**	**0.15**	**0.19**		0.05	0.07	**0.11**	**0.11**	0.09	0.09	**0.13**	**0.11**	**0.15**	0.08	0.08
Tr2								**0.13**	**0.11**	**0.11**	**0.12**	**0.10**	**0.10**	**0.11**	**0.08**	**0.09**	**0.08**	**0.08**	**0.10**	**0.16**	0.05		0.07	0.08	0.09	0.08	0.07	0.08	0.07	**0.10**	0.05	0.05
Tr3								**0.17**	**0.16**	**0.16**	**0.18**	**0.15**	**0.16**	**0.17**	**0.15**	**0.15**	**0.15**	**0.14**	**0.15**	**0.19**	0.07	0.05		0.07	**0.12**	0.08	0.09	0.09	0.09	**0.13**	0.09	0.09
Tr4								**0.14**	**0.13**	**0.13**	**0.14**	**0.12**	**0.13**	**0.13**	**0.14**	**0.13**	**0.12**	**0.10**	**0.11**	**0.14**	0.09	0.07	0.06		**0.14**	**0.11**	**0.11**	0.08	0.08	**0.12**	**0.13**	**0.12**
Ha1								**0.18**	**0.14**	**0.14**	**0.16**	**0.13**	**0.14**	**0.16**	**0.15**	**0.14**	**0.13**	**0.14**	**0.14**	**0.20**	**0.10**	0.07	**0.10**	**0.11**		0.01	0.01	**0.11**	0.09	**0.13**	0.05	0.05
Ha2								**0.18**	**0.14**	**0.13**	**0.16**	**0.12**	**0.15**	**0.15**	**0.15**	**0.14**	**0.13**	**0.13**	**0.13**	**0.20**	0.09	0.07	0.08	**0.10**	0.01		0.01	**0.10**	0.08	**0.12**	0.05	0.06
Ha3								**0.16**	**0.13**	**0.13**	**0.14**	**0.12**	**0.14**	**0.15**	**0.14**	**0.13**	**0.13**	**0.12**	**0.13**	**0.18**	0.08	0.07	0.08	**0.10**	0.01	0.01		0.09	0.06	**0.11**	0.03	0.04
Le1								**0.15**	**0.14**	**0.13**	**0.14**	**0.13**	**0.13**	**0.14**	**0.13**	**0.12**	**0.12**	**0.12**	**0.13**	**0.17**	**0.10**	0.07	0.08	0.07	0.09	0.08	0.07		0.02	0.06	**0.12**	**0.12**
Le2								**0.16**	**0.14**	**0.14**	**0.14**	**0.13**	**0.14**	**0.15**	**0.13**	**0.13**	**0.13**	**0.13**	**0.13**	**0.17**	**0.10**	0.06	0.08	0.08	0.08	0.08	0.06	0.02		0.06	**0.10**	**0.10**
Le3								**0.21**	**0.19**	**0.20**	**0.20**	**0.18**	**0.19**	**0.20**	**0.18**	**0.18**	**0.18**	**0.17**	**0.19**	**0.23**	**0.16**	**0.13**	**0.15**	**0.15**	**0.14**	**0.13**	**0.10**	0.07	0.07		**0.12**	**0.12**
Si1								**0.17**	**0.14**	**0.15**	**0.16**	**0.13**	**0.14**	**0.14**	**0.14**	**0.13**	**0.13**	**0.12**	**0.14**	**0.20**	0.06	0.05	0.08	**0.10**	0.05	0.06	0.04	**0.10**	**0.10**	**0.14**		0.00
Si2								**0.16**	**0.14**	**0.14**	**0.15**	**0.12**	**0.14**	**0.13**	**0.13**	**0.12**	**0.11**	**0.11**	**0.14**	**0.19**	0.07	0.05	0.08	0.09	0.06	0.06	0.05	**0.10**	0.09	**0.15**	0.00	

Abbraviation of each population is followed Table 2.

Values above and below the diagonals are calculated from nine loci (availabe for all species) and 12 loci (available except *A. lutchuense*), respectively.

Values above 0.1 are in bold.
