# Supplementary material for: Tight species cohesion among sympatric insular wild gingers (Asarum spp. Aristolochiaceae) on continental islands: Highly differentiated floral characteristics versus undifferentiated genotypes
Source: PLoS One. 2017 Mar 16;12(3):e0173489. doi: 10.1371/journal.pone.0173489 (PMC5354281; doi:10.1371/journal.pone.0173489)
Supplement: S5 Table — Probabilities of significant (P < 0.05) heterozygosity excess for the two-tailed sign and Wicoxon tests under the infinite allele mutation model (IAM) and stepwise mutation model (SMM) are indicated with asterisks. (PDF) [file pone.0173489.s007.pdf]

S5 Table Probability of a bottleneck effect. Probabilities of significant ($P < 0.05$) heterozygosity excess for the two-tailed sign and Wicoxon tests under the infinite allele mutation model (IAM) and stepwise mutation model (SMM) are indicated with asterisks.

Species	Population	SIGN		Wilcoxon	
		IAM	SMM	IAM	SMM
Asarum lutchuense	Lu1	0.424	0.031*	0.250	0.027*
	Lu2	0.415	0.289	0.164	0.301
	Lu3	0.445	0.110	0.164	0.049*
	Lu4	0.353	0.477	0.426	0.910
	Lu5	0.553	0.029*	0.426	0.037*
	Lu6	0.217	0.103	0.203	0.037*
	Lu7	0.428	0.040*	0.820	0.027*
A. fudsinoi	Fu1	0.017*	0.228	0.002*	0.110
	Fu2	0.015*	0.385	0.001*	0.339
	Fu3	0.233	0.070	0.008*	0.092
	Fu4	0.069	0.604	0.042*	1.000
	Fu5	0.070	0.199	0.003*	0.424
	Fu6	0.084	0.359	0.002*	0.470
A. celsum	Ce1	0.019*	0.367	0.034*	0.301
	Ce2	0.080	0.072	0.042*	0.233
	Ce3	0.082	0.372	0.005*	0.519
	Ce4	0.018*	0.595	0.001*	0.569
A. gusk	Gu1	0.002*	0.017*	0.000*	0.110
	Gu2	0.424	0.355	0.151	0.339
A. pellucidum	Pe1	0.545	0.233	0.339	0.424
A. trinacriforme	Tr1	0.040*	0.129	0.001*	0.273
	Tr2	0.053	0.251	0.027*	0.636
	Tr3	0.001*	0.254	0.002*	0.376
	Tr4	0.044*	0.524	0.027*	0.588
A. hatsushimae	Ha1	0.041*	0.271	0.027*	0.588
	Ha2	0.043*	0.519	0.001*	0.787
	Ha3	0.011*	0.450	0.021*	0.273
A. leucosepalum	Le1	0.051	0.548	0.027*	0.497
	Le2	0.057	0.009*	0.004*	0.080
	Le3	0.044*	0.120	0.005*	0.168
A. simile	Si1	0.047*	0.233	0.005*	0.497
	Si2	0.011*	0.040*	0.001*	0.127
